# Primary process emotion, identity, and culture: cultural identification's roots in basic motivation

**DOI:** 10.3389/fpsyg.2015.00218

**Published:** 2015-02-27

**Authors:** Gregory Bonn

**Affiliations:** Graduate School of Education and Human Development, Nagoya UniversityNagoya, Japan

**Keywords:** culture, emotion, attachment, motivation, cultural symbols, identity, meaning, security

## Primary process emotions, identity, and culture

The study of culture and psychology has generated a number of influential theoretical perspectives (e.g., Henrich et al., 2010; Markus and Kitayama, 2010). Less attention, however, has been directed at the psychological basis of cultural experience. What is it about cultural symbols that leads humans everywhere to create them? And, why do certain cultural symbols, be they religion, guns, food, or music hold such emotional weight for many of us? A simple answer could be “Humans can only survive in groups. We need shared symbols to communicate and cooperate with each other” This is essentially true, but here I argue that primary-process emotions identified by the field of affective neuroscience, further reflected upon in the context of several developmental theories, can elucidate the emotional nature of cultural experience. Culture, we shall see, is important not just because it teaches the skills needed to survive in a particular social and physical environment: It also satisfies a primary emotional need for security and predictability. I argue that a primitive attachment to culture goes beyond cognitive abilities and language to the very basis of human emotion and motivation. Thinking about cultural symbols in this way helps to understand the strength, and it can be argued, irrationality, of feelings and behaviors sometimes associated with them.

## Hard-wired for relationships

It has become clear that humans are endowed from birth with cognitive capacities that enable them to engage with and learn from others. From early infancy, children prefer human faces to other stimuli (Frank et al., 2009); they show interest in the attentional objects of others (Hoehl et al., 2008); and they are highly sensitive to others' emotional states (Schore, 2003). Overall, evidence suggests that young children have innate biases (i.e., genetically based cognitive modules) directing them toward absorbing certain types of knowledge: Abilities such as recognizing specific faces, learning language, understanding motion, imputing mental states to others, and grouping people according to categories develop naturally in humans everywhere (see Sperber and Hirschfeld, 2004, for a review). From the earliest stages of their lives, normally developing humans actively engage with the social world, expressly seeking out and preferentially learning from interactions with other humans. These strong tendencies to seek out other people and to absorb social information, are complemented by an, as far as we know, uniquely human degree of behavioral and cognitive flexibility. Human groups not only adapt their behaviors to different environments, but also mold those environments in many ways to suit their needs. Cultures can be thought of as human created “environmental niches” (Laland et al., 2000) held together by symbols and practices that are passed along across generations. In the context of human development these symbols and traditions become, over time, an integral part of how an individual comes to understand his own existence. As a child gradually learns to make sense of his existence in the world, his ideas about who he is and what purpose his life serves, are dependent upon his relationship to the symbols that hold society together.

Evolution has preprogrammed humans to absorb traditions from their social groups and thus to benefit from the collective experiences of their elders. Much of the pervasiveness of cultural traditions can be attributed to this: Once a group of people has figured out how to survive well enough in a given environment, younger generations naturally copy the behaviors of those around them (Quinn, 2003). Cultural traditions that are successful at producing offspring and providing for them, naturally and automatically, replicate themselves through the machinery of learning biases. Such learning tendencies, however, important and distinctively human as they are, do not explain at a deeper level the emotional significance of culture. There is a power inherent in certain cultural symbols that transcends simple learning, or even survival: People at times become more attached to cultural symbols than to life itself.

## Emotion and motivation

Learning processes and learning biases explain much about social learning and cultural transmission, but they do not fundamentally explain human motivation: They do not explain why humans actually *do* anything. If we, to some degree, accept arguments for modularity, or the relative isolation within the brain, of specific cognitive or information processing abilities (e.g., Sperber and Hirschfeld, 2007), it is important to consider what drives humans to seek out information in the first place. In other words, cognitive abilities and motivation should be thought of as complementary, but separate, domains. Something must drive humans to engage with the world and acquire the information they are so well equipped to process.

Panksepp's (2004, 2011) work in the field of affective neuroscience provides such a starting point: He has identified seven “primary process” emotional systems that have similar physiological substrates in all mammals: SEEKING, FEAR, PANIC, CARE, RAGE, LUST, and PLAY. Each system can be reliably activated through the stimulation of distinct sub-cortical brain regions in both humans and other mammals. Primary process emotions, Panksepp argues, are the motivational foundation for appetitive and self-preservatory behaviors. Primary emotional systems motivationally shape engagement with the environment and thus serve to direct learning processes: A child will not explore its environment or actively seek out novel stimuli if it is experiencing fear or panic. From earliest childhood, basic emotions provide the motivation for exploring and learning about the environment or for retreating to the relative safety of the familiar. As a simple example, when the SEEKING system of a human is activated (related to the ventral tegmental area and the nucleus accumbens) he will feel alert and expectant: Having the feeling that something good is nearby, he actively explores and seeks out positive, fitness enhancing, experiences such as food, sex, social interaction etc. On the other hand, if the FEAR system (based around the amygdala and hypothalamus) is stimulated, people feel intense anxiety, like they are being chased or their life is threatened. FEAR thus stimulates hiding or fleeing, essentially the opposite of SEEKING. Through experience, the activation of particular emotional systems become associated with various stimuli—people, places, objects, etcetera, and a basic form of symbolism takes root.

## Basic emotions and attachment

Two of Panksepp's basic emotions, CARE and PANIC, are at the core of what (Bowlby, 1969, 1988) termed the “attachment behavioral system.” CARE is the trigger of nurturing behaviors found in all mammals. PANIC is the separation anxiety or distress which keeps young mammals within safe proximity to their caregivers. Offspring treat the parent as a kind of “secure base,” moving closer when threatened or unsure. Parental CARE-related behaviors complement the PANIC system by motivating the parent to respond to various signals (e.g., cries, anxious behavior) and to reduce the offspring's anxiety. The PANIC system, Panksepp notes, is anatomically closely related to physical pain circuits. Thus, there is a strong intrinsic motivation for humans to keep caregivers close at hand: Proximity is security, it alleviates an intense anxiety, the emotional pain of being alone. Over time, a child learns which aspects of his world are safe, and desirable; what Panskepp refers to as “comfort zones.” When in a comfort zone, the SEEKING or exploratory affect system is safely activated: The child feels driven to explore, play, and thus learn more about his environment. When possible threats or uncertain stimuli are encountered, however, the FEAR system is activated, motivating the child to retreat toward his secure parental base. The qualitatively separate emotion of PANIC or anxiety is triggered by the perceived absence of a secure base; the child, in essence does not know where to turn. Even in the absence of a direct threat, the feelings of anxiety which are brought on by the absence of a secure base will tend to preempt the activation of more “positive” or approach-related emotions such as SEEKING or PLAY. The PANIC or anxiety system—in concert with the corresponding parental CARE or nurturing system, thus, serve as base level motivators that keep a child (or other young mammal) and its caregivers in close proximity. Bowlby and others (e.g., Erikson, 1950; Schore, 2003) have argued that essential characteristics of the self are forged within the dynamics of these early relationships as well as in the cultural nature of later childhood interactions (Miller et al., 1996, 1997; Quinn, 2003; Tobin et al., 2009).

## Culture as secure base

Thus, humans are hard-wired from birth to seek out safety and security in other people, and their early intuitive sense of the world is shaped by the nature of these associations. Here I argue that this fundamental emotional need for security in human relationships does not disappear as a child's conceptions of the world grow more complex. Instead, what occurs is a gradual transformation of the nature of the secure base. As a child becomes more familiar with his environment, and his internal models of how the world works become more elaborate, the symbolic role of the caregiver as the primary source of security and safety is gradually supplanted by other developmentally relevant relationships and stimuli. Developmental psychologists (e.g., Erikson, 1950; Damon and Hart, 1988) point to clear shifts in the focus of children's attention as well as their conceptualization of the self as they progress from infancy through childhood and adolescence. Through a normal developmental process, the emotional weight of the caregiver as the primary source of security, safety, and self-definition shifts to various peer groups (see also Harris, 1998) and later to more abstract symbols. Through childhood and adolescence there is a gradual expansion of symbolic safety zones related to greater knowledge of the world as well as an increased capability for abstraction. A meaningful sense of personal continuity develops (Habermas and Bluck, 2000; McAdams, 2001). A sense of history and a sense of personal themes; capabilities, and limitations, as well as likes and dislikes emerges. A symbolic sense of *identit*y (e.g., Erikson, 1968) develops. An individual creates a symbolic representation of the self-in-relation-to-society that allows him to thrive in a particular human-created environmental niche. It allows the individual to make plans and predictions about his surroundings and provides an emotional comfort zone (Minoura, 1992) in which emotions such as SEEKING, CARE, and PLAY are free to express themselves.

The social nature of identity, however, also relates to an intrinsic dark side which is often overlooked (e.g., Erikson, 1975). When there are perceived threats to the social foundations of a person's identity; when people feel like their safety zones are somehow less secure; when unfamiliar practices and ideas knock at one's door, it is threatening. Threats to important social symbols are threats to the basic sense of the self: They threaten the sense of security and safety that is necessary to interact effectively with the world. No wonder, then, that emotions like PANIC, FEAR, and RAGE become easily engaged at such times, and why history is replete with horrors committed in the name of cultural symbols. Thus, I argue here that the same primary sub-cortical emotional drive for safety, security, and predictability underlies both adults' adhesion to cultural symbols and childhood attachment behaviors. Culture is the secure base from which enculturated individuals can explore comfortably. This idea is reminiscent of Terror Management Theory (TMT; Pyszczynski et al., 1999) which has been well supported empirically over the years. A critical distinction here is that TMT conceptualizes cultural identification as a defense against a cognitive, or conscious, fear of death. If we consider human behavior from the level of basic emotions, loyalty to groups and their symbols does not require abstract knowledge of impending death. All it requires is feelings… Emotions motivate us to stay close to trusted others: We feel safe and secure when we are symbolically in the presence of trusted others and their representations. We feel anxious, angry, and hateful when that presence is threatened.

### Conflict of interest statement

The author declares that the research was conducted in the absence of any commercial or financial relationships that could be construed as a potential conflict of interest.
